# Physical Activity Levels Among Portuguese Physical and Rehabilitation Medicine Physicians: A Cross-Sectional Comparison With the General Population

**DOI:** 10.7759/cureus.107345

**Published:** 2026-04-19

**Authors:** David Cordeiro, Maria Ana Pinheiro, Gonçalo Pereira, Isabel Ponte, João Luís, Margarida Freitas

**Affiliations:** 1 Physical and Rehabilitation Medicine, Unidade Local de Saúde Almada Seixal, Almada, PRT

**Keywords:** burnout, physical activity, physical exercise, physicians, sedentary behavior

## Abstract

Introduction

Physical activity plays a vital role in maintaining health and preventing chronic diseases. Despite its well-established benefits, sedentary behavior remains highly prevalent. Physicians, as health promoters, should adopt behaviors consistent with their clinical recommendations, thereby positively influencing their patients.

Objective

The primary aim of this study was to perform an approximate, exploratory comparison of the self-reported physical activity levels of Portuguese Physical and Rehabilitation Medicine (PRM) physicians with those of the general population, as assessed by the Special Eurobarometer 525. Secondary objectives included analysis of associated factors such as career stage, employment sector, motivations, and barriers to exercise participation.

Methods

A cross-sectional observational study was conducted using an anonymous online questionnaire distributed between November and December 2024. PRM physicians practicing in Portugal, in both the public and private sectors, were included. Physical activity levels were assessed using the short form of the International Physical Activity Questionnaire (IPAQ-SF). General population data were obtained from the Special Eurobarometer 525. Descriptive statistics and chi-square tests were used for data analysis.

Results

A total of 210 physicians participated, of whom 123 (58.6%) were female, and 87 (41.4%) were male, with a median age of 38 years. Among the respondents, 80 (38.1%) reported no vigorous activity and 71 (33.8%) reported no moderate activity in the preceding week, both lower than the proportions observed in the general population (493/1006 (49.0%) and 382/1006 (38.0%), respectively). However, PRM physicians concentrated their physical activity on fewer days per week. Residents in the early stages of training reported the highest levels of physical activity. An observed downward trend was noted as career stages progressed; however, this should be interpreted with caution. This decline likely reflects a complex interplay of factors, including advancing age and shifting life-course priorities, which are inherently correlated with professional progression in this sample. No statistically significant association with employment sector was identified in this sample. The most frequently reported barrier was lack of time (n = 128; 61.0%).

Conclusions

PRM physicians in this sample reported higher proportions of engagement in vigorous and moderate physical activity compared with the general Portuguese population, although this comparison is approximate due to methodological differences between instruments. These findings suggest that challenges related to exercise regularity persist across advancing career stages. Institutional policies supporting work-life balance and physician well-being may be warranted.

## Introduction

Regular physical activity and exercise confer well-established health benefits, including the prevention and management of chronic diseases, improvement of mental health, and promotion of overall well-being. Physical activity significantly reduces the risk of cardiovascular and cerebrovascular diseases, diabetes, and other metabolic disorders, and contributes to the prevention of certain neoplasms. It also improves bone and muscle mass, enhances cognitive function, and reduces depressive symptoms, positively affecting quality of life. A comprehensive overview of Cochrane systematic reviews has demonstrated that regular physical activity is associated with a 13% reduction in all-cause mortality and improvements in both general and health-related quality of life [1].

Although the terms “physical activity” and “exercise” are frequently used interchangeably, they describe distinct concepts. According to Caspersen et al., physical activity refers to any bodily movement produced by skeletal muscle that results in energy expenditure, occurring in various contexts including occupational, domestic, recreational, or transportation settings. Exercise is a subcategory of physical activity that is planned, structured, repetitive, and designed to improve or maintain physical fitness, performance, or health [2].

Beyond its consequences for individual health, physical inactivity represents a significant cost to healthcare systems. It has been estimated that by 2030, potentially preventable conditions associated with insufficient exercise will result in a global cost of 47.6 billion dollars annually [3].

The World Health Organization (WHO) 2020 guidelines on physical activity and sedentary behavior recommend that adults engage in at least 150 minutes per week of moderate-intensity aerobic activity or 75 minutes of vigorous-intensity activity [4]. Despite this well-established and widely disseminated knowledge, sedentary behavior remains prevalent. Recent pooled analyses indicate that 31% of adults and 80% of adolescents worldwide fail to meet the minimum physical activity recommendations [5,6]. In many European countries, including Portugal, this concerning trend persists. The Special Eurobarometer 525, a survey conducted by the European Commission between April 19 and May 16, 2022, through in-person and online interviews with citizens of the 27 Member States, revealed that Portugal has one of the highest rates of sedentary behavior. According to the survey data, 73% of Portuguese respondents reported never engaging in exercise or sport, a figure substantially above the EU-27 average of 45%. Furthermore, the proportion of inactive individuals in Portugal increased by 5% relative to the previous survey conducted in 2017 [7].

One of the tools used to assess physical activity levels is the International Physical Activity Questionnaire (IPAQ), an internationally standardized instrument developed with the support of the WHO and the Centers for Disease Control and Prevention, with the goal of enabling comparisons across different populations and cultural contexts [8].

Healthcare professionals, particularly physicians, play a crucial role in promoting physical activity among their patients. Evidence suggests that physically active physicians tend to counsel their patients more frequently about the importance of exercise, thereby positively influencing their habits [9-12].

The prevention and rehabilitation of chronic diseases are central responsibilities of medical practice, and in this context, encouraging increased physical activity and exercise plays a predominant role in the clinical activity of Physical and Rehabilitation Medicine (PRM) specialists. Accordingly, the primary aim of this study was to perform an approximate comparison of the self-reported physical activity levels of PRM physicians in Portugal with those of the Portuguese general population, as assessed by the Special Eurobarometer 525. Through this analysis, we sought to contribute to a better understanding of activity habits among these healthcare professionals. Secondary objectives included assessing variation in activity levels across career stages, examining the relationship between physical activity and employment sector, describing motivations and barriers to exercise, and evaluating the influence of sport interest on specialty choice.

## Materials and methods

Study design and setting

A cross-sectional observational study was conducted targeting PRM physicians practicing in Portugal. Data were collected through an anonymous online questionnaire (Google Forms, Google, Inc., Mountain View, CA) available between November 14 and December 14, 2024. The questionnaire (Appendices) was distributed via institutional email lists of PRM departments and professional societies, as well as through social media channels. Participation was voluntary and preceded by acceptance of an informed consent form that described the study objectives, guaranteed anonymity, and outlined participants’ rights.

Inclusion and exclusion criteria

The complete inclusion and exclusion criteria are presented in Table 1.

**Table 1:** Inclusion and exclusion criteria

Criterion Type	Description
Inclusion	Physicians with active clinical practice in Physical and Rehabilitation Medicine (PRM) within Portuguese national territory
	Residents (in specialty training) or board-certified PRM specialists
	Practice in any employment sector (public, private, or mixed)
	Voluntary acceptance of the informed consent form prior to questionnaire completion
Exclusion	Physicians not currently practicing PRM in Portugal
	Incomplete questionnaire submissions (all fields required for analysis)

Data collection

The categories of information collected are summarized in Table 2. The questionnaire incorporated the short form of the IPAQ (IPAQ-SF), an internationally validated instrument designed to quantify physical activity levels in the seven days preceding its administration. Physical activity levels were classified into three categories - low, moderate, and high - in accordance with IPAQ scoring guidelines, based on the calculation of metabolic equivalent of task minutes per week (MET-minute/week). All questionnaire fields were mandatory; only fully completed submissions were included in the analysis, and no imputation of missing data was performed. Prior to statistical processing, the dataset was manually screened for potential duplicate entries and inconsistent responses (e.g., entries exceeding logical daily time limits); no such instances were found. No imputation of missing data was performed

**Table 2:** Categories of data collected IPAQ-SF = short form of the International Physical Activity Questionnaire

Category	Variables Collected
Demographic data	Age (years), sex (female, male, other), region of residence (North, Center, South, Azores, Madeira)
Professional data	Career stage (resident - 1st half of training, resident - 2nd half, specialist < 5 years, 5-10 years, 10-15 years, ≥ 15 years); employment sector (public, private, mixed)
Physical activity (IPAQ-SF)	Number of days per week with vigorous activity, moderate activity, and walking (≥ 10 consecutive minutes); average daily duration of each; daily sitting time
Sport practice	Regular sport participation (yes/no), sport modality(ies), registered competitive athlete status (yes/no)
Motivations and barriers	Self-reported motivations for physical activity (multiple-choice); self-reported barriers to regular exercise (multiple-choice)
Specialty choice	Self-reported influence of sport interest on the decision to pursue PRM as a medical specialty (yes/no)

Sample size considerations 

According to data provided by the Portuguese Medical Association (Ordem dos Médicos), as of May 2025, 774 PRM specialists were registered in Portugal. Considering an average of approximately 40 residents admitted annually and a training duration of five years, an estimated 200 residents were in training, yielding a total target population of approximately 974 PRM physicians (specialists and residents). For a finite population of this size, 210 responses correspond to a margin of error below 6.5% at a 95% confidence level. No formal a priori sample size calculation was performed, as the study aimed to survey the entire accessible population; the achieved sample of 210 participants (approximately 21.6% of the estimated workforce) was therefore a convenience sample, and its representativeness should be interpreted with caution given the voluntary, non-random recruitment strategy.

Statistical analysis

Statistical analysis was performed using Microsoft Excel (version 365; Microsoft Corporation, Redmond, WA) and IBM SPSS (version 30.0.0; IBM Corp., Armonk, NY). The distribution of continuous variables was assessed using the Kolmogorov-Smirnov test. As age did not follow a normal distribution (p < 0.05), it was reported as median with interquartile range (IQR). Categorical variables, including sex, fracture type, and comorbidities, were reported as absolute frequencies and percentages.

For comparison with the Portuguese general population, data from the Special Eurobarometer 525 were used. Because the Eurobarometer questionnaire incorporates questions structurally similar - but not identical - to the IPAQ-SF, and because its data are released only in aggregate form (precluding individual-level MET-minute/week calculations), the comparison was performed using proportions of individuals who reported each physical activity frequency category (vigorous and moderate). This comparison is therefore approximate and exploratory and should not be interpreted as a direct equivalence between instruments.

Associations between physical activity levels, career stage, and employment sector, as well as comparisons with the general population, were assessed using the chi-square (χ²) test. The significance level was set at p < 0.05. A 95% confidence interval (95% CI) was calculated for the overall prevalence of physical activity engagement in the sample. Where applicable, Cramér’s V was calculated as a measure of effect size for chi-square tests. The analysis was exclusively bivariate; no multivariable adjustment for potential confounders (age, sex, workload, family responsibilities) was performed, which is acknowledged as a limitation of the study.

Ethical considerations

The study was conducted in accordance with the principles of the Declaration of Helsinki and received a favorable opinion from the Ethics Committee of the Unidade Local de Saúde Almada-Seixal (approval number 67/2024). Informed consent was obtained from all participants prior to their inclusion.

## Results

Of the 210 physicians who completed the questionnaire, 123 (58.6%) were female, and 87 (41.4%) were male. The median age was 38 years (IQR: 31-47; range: 26-75 years). The majority of participants resided in the Northern region of Portugal (n = 85; 40.5%) (Table 3).

**Table 3:** Demographic and professional characteristics of the study sample Age is reported in years. IQR = interquartile range

Variable	Total (n = 210)
Age (years), median (IQR)	38 (31-47)
Sex	
Female	123 (58.6%)
Male	87 (41.4%)
Region of residence	
North	85 (40.5%)
Center	38 (18.1%)
South	76 (36.2%)
Azores	8 (3.8%)
Madeira	3 (1.4%)
Career stage	
Resident - 1st half of training	36 (17.1%)
Resident - 2nd half of training	35 (16.7%)
Specialist (< 5 years)	33 (15.7%)
Specialist (5-10 years)	21 (10.0%)
Specialist (10-15 years)	31 (14.8%)
Specialist (≥ 15 years)	54 (25.7%)
Employment Sector	
Public	53 (25.2%)
Private	45 (21.4%)
Mixed (public and private)	112 (53.3%)

Participants were grouped according to career stage and employment sector. Career stage categories included residents in the first half of training (n = 36; 17.1%), residents in the second half of training (n = 35; 16.7%), specialists with less than five years of practice (n = 33; 15.7%), five to 10 years (n = 21; 10.0%), 10 to 15 years (n = 31; 14.8%), and 15 or more years (n = 54; 25.7%). The majority of respondents worked in a mixed setting (public and private) (n = 112; 53.3%), followed by the public sector exclusively (n = 53; 25.2%) and the private sector (n = 45; 21.4%) (Table 3).

Regarding physical activity levels in the preceding week, 75 physicians (35.7%; 95% CI: 29.2-42.2%) were classified as having a moderate level, 71 (33.8%; 95% CI: 27.3-40.3%) a high level, and 64 (30.5%; 95% CI: 24.0-37.0%) a low level, according to IPAQ-SF scoring criteria.

Approximate comparison with the general population

Comparison with the Portuguese general population revealed a statistically significant difference in the distribution of vigorous physical activity frequency (χ² = 17.63; p < 0.001; Cramér’s V = 0.12). Among PRM physicians, 80 (38.1%) reported no vigorous activity in the preceding week, a proportion lower than that observed in the general population (n = 493; 49.0%). Ninety-two physicians (43.8%) engaged in vigorous activity on one to three days per week, compared with 292 (29.0%) in the general population. However, only 38 (18.1%) of PRM physicians did so on four or more days, versus 221 (22.0%) in the general population (Table 4).

**Table 4:** Comparison of weekly physical activity frequency between PRM physicians and the general Portuguese population Statistical significance was set at p < 0.05. PRM = Physical and Rehabilitation Medicine

Weekly Frequency	PRM Physicians (n = 210)	General Population (n = 1006)
Vigorous activity		
0 days	80 (38.1%)	493 (49.0%)
1-3 days	92 (43.8%)	292 (29.0%)
4-7 days	38 (18.1%)	221 (22.0%)
Chi-square (χ²)		17.63
p-value		< 0.001
Cramér's V		0.12
Moderate activity		
0 days	71 (33.8%)	382 (38.0%)
1-3 days	118 (56.2%)	403 (40.1%)
4-7 days	21 (10.0%)	211 (21.0%)
Do not know	0 (0%)	10 (1.0%)
Chi-square (χ²)		24.41
p-value		< 0.001
Cramér's V		0.14

A statistically significant difference was also observed in the distribution of moderate physical activity frequency (χ² = 24.41; p < 0.001; Cramér’s V = 0.14). Seventy-one physicians (33.8%) reported no moderate activity in the preceding week, slightly lower than the general population figure (n = 382; 38.0%). The majority of PRM physicians (n = 118; 56.2%) engaged in moderate activity on one to three days per week, compared with 403 (40.1%) in the general population, yet only 21 physicians (10.0%) reported moderate activity on four to seven days per week, below the 211 (21.0%) observed in the Portuguese population (Table 4). These findings should be interpreted with caution, as the comparison is indirect and methodologically approximate, given the differences between IPAQ-SF scoring and the aggregated Eurobarometer reporting format.

Physical activity by career stage and employment sector

Analysis of physical activity levels according to career stage revealed statistically significant differences (χ² = 26.23; p = 0.003; df = 10). Residents in the first half of training were the most active group, with 19 (52.8%) of 36 physicians at this stage reporting high activity levels; only four (11.1%) reported low levels. A declining proportion of individuals with high physical activity was observed across advancing career stages. Among specialists with 15 or more years of experience, only nine (16.7%) reported high physical activity, whereas 27 (50.0%) reported low levels according to IPAQ-SF classification (Table 5).

**Table 5:** Physical activity levels by career stage and employment sector Statistical significance was set at p < 0.05.

Category	Low n (%)	Moderate n (%)	High n (%)	Total
Career stage				
Resident - 1st half	4 (11.1%)	13 (36.1%)	19 (52.8%)	36
Resident - 2nd half	9 (25.7%)	13 (37.1%)	13 (37.1%)	35
Specialist (< 5 yr)	9 (27.3%)	9 (27.3%)	15 (45.5%)	33
Specialist (5-10 yr)	5 (20.8%)	13 (54.2%)	6 (25.0%)	24
Specialist (10-15 yr)	10 (35.7%)	9 (32.1%)	9 (32.1%)	28
Specialist (≥ 15 yr)	27 (50.0%)	18 (33.3%)	9 (16.7%)	54
Total	64	75	71	210
χ² = 26.23; p = 0.003 (df = 10)				
Employment sector				
Public	13 (24.1%)	22 (40.7%)	19 (35.2%)	54
Private	16 (36.4%)	12 (27.3%)	16 (36.4%)	44
Mixed	35 (31.3%)	41 (36.6%)	36 (32.1%)	112
Total	64	75	71	210
χ² = 2.74; p = 0.602 (df = 4)				

It should be noted that career stage is closely correlated with age and potentially with other life-course factors (e.g., family responsibilities, cumulative workload); in the absence of multivariable adjustment, these results should be interpreted as descriptive associations rather than independent effects of career progression.

No statistically significant association between physical activity levels and employment sector was identified in this sample (χ² = 2.74; p = 0.602; df = 4). Physicians in the public, private, and mixed sectors showed similar distributions of physical activity (Table 5).

Walking frequency and sedentary behavior

Regarding the frequency of walking for at least 10 consecutive minutes in the preceding week, the median was four days. Twenty-six physicians (12.4%) reported not walking for 10 or more minutes on any day, while the majority of those who did (n = 114; 62.0%) indicated a daily duration of less than 30 minutes. Concerning time spent sitting, the majority (n = 98; 46.7%) reported remaining in a seated position for more than six hours and 30 minutes daily. While there is no universally agreed threshold for “excessive” sedentary time, recent evidence suggests that occupational sitting exceeding approximately six hours per day is associated with increased cardiovascular and all-cause mortality risk, even among physically active individuals (Table 6) [13].

**Table 6:** Walking frequency and sedentary behavior among Physical and Rehabilitation Medicine physicians

Variable	Category	n (%)
Weekly frequency of walks ≥10 minutes	0 days	26 (12.4%)
	1-3 days	71 (33.8%)
	4-7 days	113 (53.8%)
Mean daily walking duration	< 30 minutes	114 (62.0%)
	≥ 30 minutes	70 (38.0%)
Daily seated time	≤ 5 hours 30 minutes	55 (26.2%)
	5 hours 31 minutes to 6 hours 30 minutes	42 (20.0%)
	> 6 hours 30 minutes	98 (46.7%)
	Do not know	15 (7.1%)

Motivations, barriers, and sport practice

The primary motivations reported for exercise were improving physical fitness (n = 164; 78.1%), improving health (n = 160; 76.2%), and relaxation (n = 117; 55.7%). The main barrier identified was lack of time (n = 128; 61.0%) (Table 7).

**Table 7:** Motivations and barriers to physical exercise among Physical and Rehabilitation Medicine physicians

Category	Item	n (%)
Motivations	Improving physical fitness	164 (78.1%)
	Improving health	160 (76.2%)
	Relaxation	117 (55.7%)
	Improving body image	101 (48.1%)
	Weight management	98 (46.7%)
	Fun	88 (41.9%)
	Counteracting effects of aging	87 (41.4%)
	Improving self-esteem	67 (31.9%)
	Spending time with friends	35 (16.7%)
	Competitive spirit	32 (15.2%)
	Developing new skills	26 (12.4%)
	Meeting people	6 (2.9%)
	Social integration	5 (2.4%)
	Do not know/no response	9 (4.3%)
Barriers	Lack of time	128 (61.0%)
	Lack of motivation or interest	29 (13.8%)
	Lack of companionship	15 (7.1%)
	Illness or disability	11 (5.2%)
	Fear of injury	10 (4.8%)
	Excessive cost	4 (1.9%)
	Lack of infrastructure	3 (1.4%)
	Fear of discrimination by other practitioners	1 (0.5%)
	Do not know/no response	4 (1.9%)

One hundred and one physicians (48.1%) reported regularly practicing at least one sport, with running being the most frequently cited (n = 32; 15.2%). Among practitioners, 17 (16.8%) were registered competitive athletes. Of note, 124 (59.0%) of the physicians surveyed stated that their interest in sport influenced their choice of the PRM specialty (Table 8).

**Table 8:** Sport practice and influence on specialty choice

Category	Subcategory	n (%)
Sport practice	Practitioners	101 (48.1%)
	Non-practitioners	109 (51.9%)
Sports modalities	Running	32 (31.7%)
	Padel	23 (22.8%)
	Swimming	12 (11.9%)
	Football/futsal	10 (9.9%)
	CrossFit	9 (8.9%)
	Gymnastics	8 (7.9%)
	Tennis	8 (7.9%)
	Martial arts	6 (5.9%)
	Cycling	6 (5.9%)
	Surf/bodyboard	5 (5.0%)
Registered competitive athletes	Yes	17 (16.8%)
	No	84 (83.2%)
Influence of sport on specialty choice	Yes	124 (59.0%)
	No	86 (41.0%)

## Discussion

The results of this study suggest that Portuguese PRM physicians may report higher proportions of engagement in vigorous and moderate physical activity compared with the Portuguese general population, as assessed by the Special Eurobarometer 525. Whereas 493 (49.0%) of the general population reported no vigorous physical activity on any day of the preceding week, only 80 (38.1%) of PRM physicians reported this level of inactivity. This trend may reflect a greater awareness of the benefits of physical activity, consistent with the professional profile of the participants, whose clinical practice is closely related to health promotion and exercise. However, it is important to emphasize that this comparison is indirect and approximate, given the methodological differences between the IPAQ-SF (used in the present study) and the aggregated format of the Eurobarometer data. Additionally, voluntary participation may have introduced selection bias favoring more physically active physicians, and the findings should therefore be interpreted as exploratory rather than definitive.

Despite the higher overall proportions of physicians reporting any vigorous or moderate physical activity, these professionals tended to concentrate their exercise on fewer days per week. While a higher proportion of physicians engaged in activity at least once weekly, the general population displayed a more frequent distribution (≥4 days). This pattern may reflect time constraints and difficulties in personal scheduling, a finding corroborated by the fact that the primary reported barrier to physical activity was lack of time (n = 128; 61.0%). This observation is consistent with prior studies highlighting professional overload as a barrier to regular physical activity among healthcare workers [14].

The concentration of physical activity on fewer days may have potential implications for injury risk. This phenomenon, commonly described as the “weekend warrior” pattern, is characterized by intense exercise sessions performed on only one or two days per week. Although recent evidence suggests that this pattern confers cardiovascular and mortality benefits similar to those observed in regularly active individuals - provided the total weekly volume meets WHO recommendations [15] - the concentration of physical load within short periods has been associated with an increased risk of musculoskeletal injuries. Gabbett demonstrated that abrupt variations in training load are among the principal predictors of injury, even among physically active individuals [16]. However, the present study did not directly assess injury outcomes, and this interpretation remains speculative.

The relationship between career stage and physical activity was also evident, with a statistically significant association observed (χ² = 26.23; p = 0.003). Residents in the first half of training were the most physically active group, which may reflect greater time availability, lower clinical workload, or higher personal motivation. A declining trend in physical activity was observed across advancing career stages, with specialists practicing for more than 15 years constituting the group with the highest proportion of individuals classified as having low physical activity according to IPAQ-SF criteria. This pattern may be related to the progressive increase in professional and family responsibilities. Importantly, career stage is closely correlated with age, and without multivariable adjustment for potential confounders such as age, sex, family status, and total working hours, the independent contribution of career progression to physical activity levels cannot be established from these data.

No statistically significant association between physical activity levels and employment sector was identified in this sample (χ² = 2.74; p = 0.602). This null finding should be interpreted cautiously; the absence of a statistically significant association in this sample does not necessarily indicate that the workplace setting has no influence on physical activity habits. Other factors, such as individual motivation, time management, and personal circumstances, may be more relevant determinants.

Regarding sedentary behavior, 140 (66.7%) of physicians reported sitting for more than five hours and 30 minutes per day, a finding that warrants attention given emerging evidence linking prolonged occupational sitting to adverse cardiovascular and metabolic outcomes, even among physically active individuals [13]. This observation underscores the potential value of institutional strategies aimed at reducing sedentary time among healthcare professionals.

Concerning exercise motivation, physicians cited physical fitness improvement and health enhancement as their top priorities, reflecting a positive perception of the impact of physical activity on quality of life. The self-reported influence of sport interest on specialty choice (reported by 124 (59.0%) of physicians) reinforces the affinity between athletic practice and professional vocation in PRM.

Strengths and limitations

This study has several strengths. The sample of 210 participants represents approximately 21.6% of the estimated PRM physician workforce in Portugal (approximately 974 physicians), enabling an analysis with a margin of error below 6.5% at a 95% confidence level. However, given the voluntary and non-random recruitment strategy (institutional email and social media), the sample cannot be considered strictly representative, and selection bias - potentially favoring physicians with greater interest in physical activity or technology - cannot be excluded. An additional strength lies in the use of an internationally validated instrument (IPAQ-SF) for standardized classification of physical activity levels.

Several limitations must be acknowledged. First, the comparison with the general population is indirect and approximate, as the Eurobarometer 525 publishes only aggregated data that do not permit individual-level MET-minute/week calculations. Although the Eurobarometer questionnaire includes items structurally similar to the IPAQ-SF, the two instruments are not identical, and the comparison should be regarded as contextual and exploratory rather than as a direct equivalence. Second, the cross-sectional design precludes causal inferences; the observed associations between career stage and physical activity may reflect confounding by age, family responsibilities, or cumulative professional burden rather than an independent effect of career progression. Third, the self-reported nature of the IPAQ-SF may introduce recall bias or social desirability bias, potentially leading to overestimation of activity levels. Fourth, the statistical analysis was exclusively bivariate; the absence of multivariable adjustment for potential confounders (age, sex, workload, family responsibilities) limits interpretation, particularly regarding the observed decline across career stages. Finally, longitudinal studies would be needed to determine whether the observed cross-sectional patterns reflect true within-individual changes in physical activity over the course of a medical career.

## Conclusions

In this cross-sectional study of 210 Portuguese PRM physicians, the data suggest that a higher proportion of physicians reported engagement in vigorous and moderate physical activity compared with the general Portuguese population, although this comparison is approximate due to methodological differences between instruments. Physical activity was concentrated on fewer days per week and appeared to decline across advancing career stages, though this finding may be confounded by age and other life-course factors. No association between employment sector and physical activity levels was identified in this sample. Lack of time was the primary barrier reported, reflecting the impact of clinical workload on exercise regularity.

Given the fundamental role of PRM physicians in promoting healthy lifestyles, these findings highlight the potential importance of institutional interventions and health policies that foster work-life balance and protect physician well-being. Prospective, longitudinal studies with multivariable adjustment are warranted to better characterize physical activity trajectories across the medical career and to evaluate the effectiveness of workplace-based interventions in this population.

## Appendices

Study questionnaire (English translation)

Original questionnaire administered in Portuguese via an anonymous online form (Google Forms). Physical activity questions were adapted from the short form of the International Physical Activity Questionnaire (IPAQ-SF).

Section 1: Informed Consent

Study Title: Assessment of Physical Activity Levels Among Physical and Rehabilitation Medicine Physicians

Principal Investigator: David Oliveira Cordeiro, PRM Resident

Study Objective: To assess the physical activity levels of PRM physicians, compare them with the general population, and describe the main activities and sports practiced.

Consent: By clicking “Yes, I agree to participate” below, you acknowledge that you understand the nature of the study, the procedures, and that you consent to participate voluntarily.

Procedures: You will be asked to complete an anonymous online questionnaire lasting approximately 5 minutes.

Confidentiality: All responses will remain anonymous and confidential.

Right to Withdraw: You may withdraw at any time, without any prejudice or need for justification, by contacting the investigator via email.

Q1. Do you agree to participate in this study?

Options: Yes, I agree to participate/No

Section 2: Demographic Data

Q2. Age

Q3. Sex

Options: Female/Male/Other

Q4. Region of Residence

Options: North/Center/South/Azores/Madeira

Q5. Career Stage

Options: Resident - 1st half of training/Resident - 2nd half of training/Specialist (< 5 years) /Specialist (5-10 years) /Specialist (10-15 years) /Specialist (≥ 15 years)

Q6. Employment Sector

Options: Public/Private/Public + Private

Section 3: Sport Practice

Q7. Do you practice any sport?

Options: Yes/No

Section 4: Sport Activity

Q8. Which sport(s) do you practice? (multiple options allowed)

Q9. Are you a registered competitive athlete?

Options: Yes/No

Q10. If yes, which sport?

Section 5: Physical Activity Level (adapted from IPAQ-SF)

Q11. In the last 7 days, on how many days did you perform vigorous physical activity (e.g., heavy lifting, running, fast cycling)?

Options: 1 day/2 days/3 days/4 days/5 days/6 days/7 days/I did not perform any vigorous physical activity in the last 7 days

Q12. If you performed vigorous activity in the last 7 days, on average, how long per day?

Q13. In the last 7 days, on how many days did you perform moderate physical activity (e.g., light lifting, low-intensity running, cycling at a leisurely pace, a doubles tennis match)?

Options: 1 day/2 days/3 days/4 days/5 days/6 days/7 days/I did not perform any moderate physical activity in the last 7 days

Q14. If you performed moderate activity in the last 7 days, on average, how long per day?

Q15. In the last 7 days, on how many days did you walk for at least 10 consecutive minutes?

Options: 1 day/2 days/3 days/4 days/5 days/6 days/7 days/I never walked for 10 consecutive minutes on any day

Q16. On the days you walked for at least 10 consecutive minutes, on average, how long did you walk?

Q17. How much time do you spend sitting on a typical day? (This should include time spent at a desk, sitting on a couch, visiting friends, studying, watching television, etc.)

Section 6: Motivations and Barriers

Q18. Why do you engage in physical activity or practice a sport? (You may select more than one option)

Options: To improve health/To improve physical fitness/To relax/To have fun/To improve athletic performance/To control body weight/To improve body image/To spend time with friends/To counteract the effects of aging/To improve self-esteem/To develop new skills/For the competitive spirit/To meet new people/To meet people from other cultures/To better integrate into society/Do not know/Other

Q19. What are the main reasons that currently prevent you from practicing sport or physical activity more regularly? (You may select more than one option)

Options: Lack of time/Lack of motivation or interest/Illness or disability/Too expensive/Does not enjoy competitive activities/Fear of injury/No adequate sports facilities nearby/No friends to practice with/Feels discriminated against by other participants/Already practices sport/physical activity regularly/Do not know/Other

Q20. Did your interest in sport influence your choice of the PRM specialty?

Options: Yes/No
